# Assessment of Concordance between 22C3 and SP142 Immunohistochemistry Assays regarding PD-L1 Expression in Non-Small Cell Lung Cancer

**DOI:** 10.1038/s41598-017-17034-5

**Published:** 2017-12-05

**Authors:** Haipeng Xu, Gen Lin, Cheng Huang, Weifeng Zhu, Qian Miao, Xirong Fan, Biao Wu, Xiaobing Zheng, Xiandong Lin, Kan Jiang, Dan Hu, Chao Li

**Affiliations:** 1 Department of Thoracic Oncology, Fujian Cancer Hospital, Fujian Medical University Cancer Hospital, Fuzhou, 350014 China; 2 Department of Pathology, Fujian Cancer Hospital, Fujian Medical University Cancer Hospital, Fuzhou, 350014 China; 3 Department of Molecular Pathology, Fujian Cancer Hospital, Fujian Medical University Cancer Hospital, Fuzhou, 350014 China; 4 Fujian Provincial Key Laboratory of Translational Cancer Medicine, Fuzhou, 350014 China; 5 Department of Pathology, School of Basic Medical Sciences, Fujian Medical University, Fuzhou, 350014 China

## Abstract

Different anti-PD-1 and anti-PD-L1 antibodies bind different epitopes. However, whether the results from the SP142 and 22C3 immunochemistry (IHC) assays can be interchanged to determine patient eligibility for immunotherapy remains largely unknown. Histologic sections from 135 tumor samples were probed with both 22C3 and SP142 antibodies. The concordance of PD-L1 expression determined by the two assays was assessed. Additionally, we evaluated the association of PD-L1 expression detected by different assays with clinicopathological features and prognosis. In total, 105 (77.78%) of 135 samples evaluated by the 22C3-IHC platform produced the same results with the SP142-IHC platform (Kappa value: 0.481, p < 0.001). In addition, 69 (51.11%) of 135 samples evaluated by the SP142-IHC platform produced the same results with the 22C3-IHC platform (Kappa value: 0.324, p < 0.001). PD-L1 expression based on the 22C3-IHC assay was significantly correlated with smoking status, whereas that based on the SP142-IHC assay was correlated with smoking status, sex, and histology. Compared to the SP142-IHC assay, the 22C3-IHC assay usually resulted in an underestimation of PD-L1 expression in tumor cells and immune cells. Thus, the results from the two assays cannot be interchanged. Our data also suggest that the use of different reagents may account for inconsistencies in the literature regarding the association between PD-L1 expression and clinicopathological features.

## Introduction

Programmed death ligand-1 (PD-L1) expression is a major immune-suppressive mechanism that is stimulated by the engagement of the PD-1/PD-L1 axis in non-small cell lung cancer (NSCLC). Tumor cells (TCs) can evade immune responses through the upregulation of PD-L1 and blockade of the PD-1/PD-L1 interaction via monoclonal antibodies and can produce a durable clinical response in patients with NSCLC. The anti-PD-1 antibody pembrolizumab and the anti-PD-L1 antibody atezolizumab are now approved by the US Food and Drug Administration (FDA) for treating patients with advanced NSCLC^1,2^.

There is data indicating that the PD-L1 expression status might help guide therapy^3,4^. For example, the results from the phase II/III KEYNOTE-010 study showed that compared with docetaxel, pembrolizumab prolonged overall survival (OS) in previously treated advanced NSCLC patients with positive PD-L1 expression determined by immunohistochemistry (IHC) on at least 1% of TCs^5^. Data from patients with advanced NSCLC in a phase III study (KEYNOTE-024) demonstrated an objective response rate (ORR) with pembrolizumab in 44.8% of patients with at least 50% PD-L1-positive TCs compared with the ORR of 27.8% with chemotherapy. The median progression-free survival (PFS) was 10.3 months in the pembrolizumab group versus 6.0 months in the chemotherapy group^6^. Based on these data, pembrolizumab was approved for use in conjunction with a companion diagnostic, the Dako 22C3 PD-L1-IHC platform. In the phase II POPLAR study, previously treated NSCLC patients (in a second- or third-line setting) were stratified by PD-L1 expression on tumor-infiltrating immune cells (ICs) and TCs assessed by IHC with the SP142 antibody and were randomized to receive atezolizumab or docetaxel treatment. Improved efficacy with atezolizumab was observed with increasing PD-L1 expression. Additionally, in the subgroup of patients with the highest PD-L1 expression (TC3 or IC3, 16% of enrolled patients), the OS was 15.5 months and 11.1 months (hazard ratio (HR) 0.46, p = 0.070), the PFS was 7.8 months and 3.9 months (HR 0.57), and the ORR was 38% and 13% for atezolizumab and docetaxel, respectively. In contrast, patients with the lowest PD-L1 levels (TC0 and IC0, 32% of patients) did not appear to benefit from atezolizumab^7^.

Currently, clinical trials have utilized different PD-L1 assays and testing platforms^8^, and various commercially available anti-PD-L1 antibodies have been used to determine PD-L1 expression in TCs and/or ICs^9^. This raises the following questions: Can different assays yield identical results for maximizing the therapeutic benefit and avoiding unnecessary toxicities? Is the use of different reagents one of the reasons for inconsistent results reporting the association between PD-L1 expression and clinicopathological features?

To better understand the differences in the results of two IHC assays for PD-L1, we assessed the conformance of PD-L1 expression between the 22C3-IHC and SP142-IHC assay results in surgically resected tumors from NSCLC patients. Moreover, we investigated the association between clinicopathologic characteristics and PD-L1 expression measured by the two assays.

## Patients and Materials

All samples were obtained with signed consent for the use of tissues under an approved protocol from the Ethical Committee of Fujian Cancer Hospital. All patients provided written informed consent for the study. All procedures performed in studies involving human participants were in accordance with the ethical standards of the institutional and/or national research committee and with the 1964 Declaration of Helsinki and its later amendments or comparable ethical standards. All experiments were performed in accordance with relevant guidelines and regulations.

We retrospectively collected 135 samples from patients with pathologically confirmed adenocarcinoma or squamous cell carcinoma at the Fujian Cancer Hospital in China. All surgeries were conducted between January 2008 and December 2010. These patients were not treated with PD-L1 axis therapies or targeted therapy. The pathological TNM stage was reassigned according to the 8^th^ TNM staging system^10^, and lung tumor histology was recategorized according to the 2015 World Health Organization (WHO) classification system for lung tumors^11^.

### PD-L1 Immunohistochemistry

Each tumor sample was pathologically examined using six 5-µm serial sections that were sent to two institutions for analysis as follows: three sections were sent to the University of Hong Kong for analysis with a 22C3-IHC on an Autostainer Link 48 with the murine 22C3 anti-human PD-L1 antibody (Code SK006, Merck & Co, Inc, Hong Kong) according to the manufacturer’s protocol – one section was stained with hematoxylin and eosin (HE); another section was stained for PD-L1; and a third section was used as the negative control. The other three sections were sent to Fujian Cancer Hospital for IHC analysis using the SP142 antibody on the Ventana Benchmark platform. Two board-certified pathologists (Chao Li and W Zhu) jointly evaluated all stained slides for PD-L1 membrane staining. All areas in each tissue section were observed for the appropriate evaluation of the expression of PD-L1 on TCs as an estimate of the percentage of TCs exhibiting partial or complete membranous staining and on ICs as an estimate of the percentage of the tumor area occupied by PD-L1-expressing ICs. Determination of PD-L1 expression was performed without considering any cut-off value, and the staining intensity was not part of this evaluation.

### PD-L1 Expression Scores and Cut-off Values

Scoring was performed based on the standards for the 22C3-IHC and SP142-IHC assays. For the 22C3-IHC assay, PD-L1 expression was determined by using a Tumor Proportion Score (TPS), which is the percentage of viable TCs showing partial or complete membrane staining. PD-L1 expression was scored at three levels, namely, TPS < 1%, TPS 1 to 49% and TPS ≥ 50%. For the SP142-IHC assay, baseline PD-L1 expression was scored using the number of PD-L1 TCs as a percentage of total TCs, as follows: TC3 ≥ 50%, TC2 ≥ 5% and < 50%, TC1 ≥ 1% and < 5%, and TC0 < 1%. Additionally, tumor-infiltrating ICs were expressed as the percentage of the tumor area as follows: IC3 ≥ 10%, IC2 ≥ 5% and < 10%, IC1 ≥ 1% and < 5%, and IC0 < 1%. To compare the overall agreement of the two assays, we interchanged various cut-off values on each set of slides stained by the two assays.

### Statistical Analysis

The weighted kappa (κ) coefficient and McNemar-Bowker test were used to evaluate the agreement and inconsistency between the results using each assay. A κ coefficient of 0.75 or less indicated poor to fair agreement, and a value greater than 0.75 indicated almost perfect agreement. Additionally, clinicopathological characteristics of PD-L1-positive tumors according to the interpretation of the 22C3-IHC or the SP142-IHC assay standards were compared with those of PD-L1-negative tumors using the χ^2^ test. The OS of patients with PD-L1-positive and PD-L1-negative tumors was estimated using the Kaplan–Meier method, and differences were compared using the log-rank test. All tests were two-sided. Statistical significance was set at p < 0.05. Statistical analyses were performed using SPSS 21.0 software.

## Results

### Patient Characteristics

A total of 135 patients were eligible for the study, and their characteristics are summarized in Table 1. Ninety-eight of the 135 patients were male (72.6%). The ECOG performance status was 0 in all patients. Adenocarcinoma and squamous carcinoma were the major histologic subtypes, accounting for 39.3% and 49.6%, respectively. One hundred and twenty patients (96.3%) had stage I-III disease, and 15 (3.7%) had stage IV disease.

**Table 1:** Clinicopathological Characteristics of Patients.

**Patient demographics (** ***N*** ** = 135)**		***N*** **(%)**
**Age**	>60 y	62 (26.3%)
	≤60 y	83 (73.7%)
**Sex**	Male	98 (72.6%)
	Female	37 (27.4%)
**Smoking status**	Never smoker	72 (55.8%)
	Former/Current smoker	57 (44.2%)
**Histology**	Squamous carcinoma	53 (39.3%)
	Adenocarcinoma	67 (49.6%)
	Other pathological types	15 (11.1%)
**TNM stage**	I	62 (45.9%)
	II	27 (20.0%)
	III	41 (30.4%)
	IV	5 (3.7%)

### Comparison of PD-L1 Expression Results Based on the 22C3-IHC and SP142-IHC Assays

**Figure 1:**
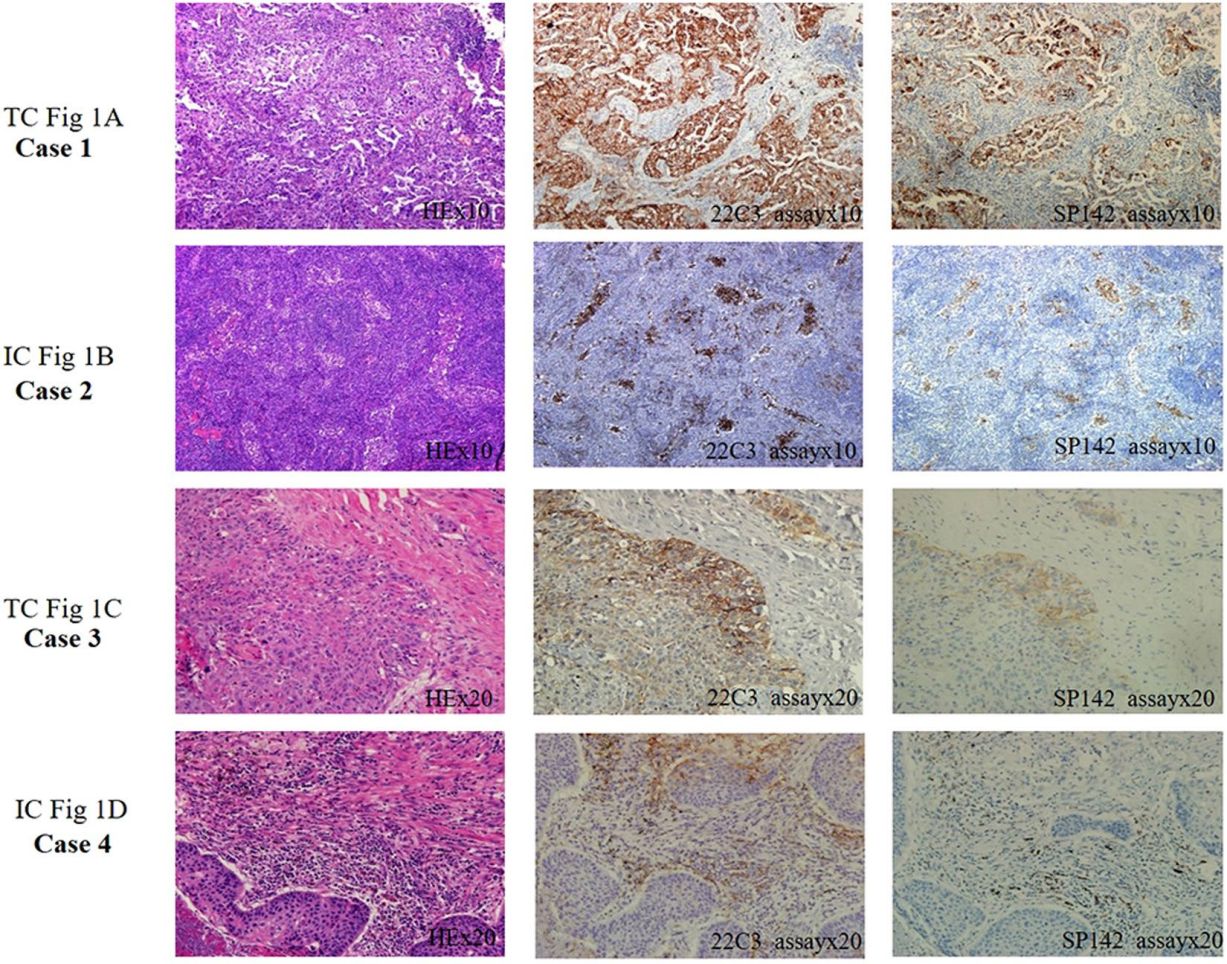
Representative micrographs of two PD-L1 IHC assays for TCs or ICs. (**A**) 22C3-IHC (80%) and SP142-IHC (70%) assays indicating strong PD-L1-positivity in TCs; (**B**) 22C3-IHC (10%) and SP142-IHC (10%) assays indicating strong PD-L1-positivity in ICs; (**C**) 22C3-IHC (30%) and SP142- IHC (5%) assays indicating weak PD-L1-positivity in TCs; and (**D**) 22C3-IHC (10%) assay indicating strong PD-L1-positivity in ICs, whereas SP142-IHC assay indicative negative staining in ICs.

**Figure 2:**
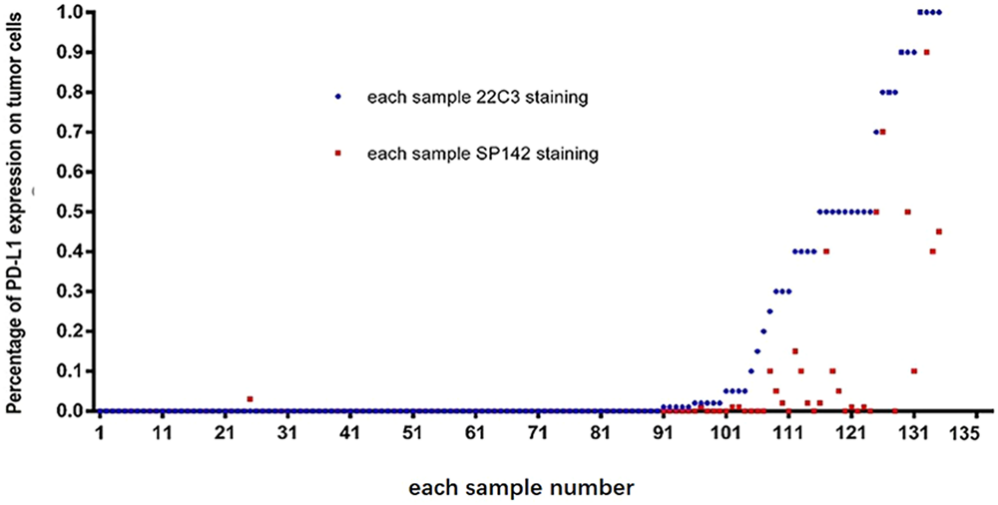
Difference between the two assays for TCs in each sample.

**Figure 3:**
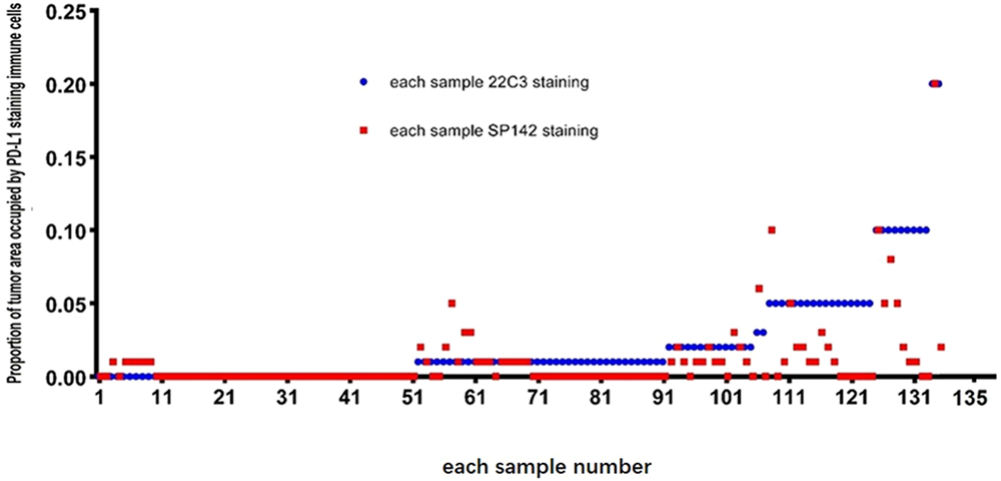
Difference between the two assays for ICs in each sample.

PD-L1 staining was observed in both TCs and ICs by both 22C3-IHC and SP142-IHC assays. Representative examples of PD-L1 staining in TCs and ICs based on two assays are shown in Fig. 1. Compared with the 22C3-IHC assay, the SP142-IHC assay resulted in weaker staining of TC membranes and fewer positive TCs (Fig. 2 **)**. One sample demonstrated 1% PD-L1-positive TCs based on the SP142-IHC assay, but it was negative based on the 22C3-IHC assay. The variability in the staining pattern of ICs between samples was greater than that for TCs **(**Figs 2 and 3 **)**. In a total of 62 discordant samples, compared to the 22C3-IHC assay, the SP142-IHC assay resulted in the underestimation and overestimation of PD-L1 expression in ICs in 53 (39.2%) and 9 (6.7%) samples, respectively (*p* < 0.001). With the adoption of the 22C3-IHC assay scoring algorithm for TCs, 20 (14.8%), 25 (18.5%), and 90 (66.7%) of the 135 samples were classified into groups that were strongly positive, weakly positive, and negative for PD-L1, respectively. Moreover, 105 (77.78%) of 135 samples produced the same results with the SP142-IHC reagent assay (κ value: 0.481, *p* < 0.001). The PD-L1 levels on TCs as detected by the 22C3-IHC assay were underestimated by the SP142-IHC assay in 29 samples. Only one sample with negative PD-L1 expression based on the 22C3-IHC assay was misclassified as having weak positive PD-L1 expression based on the SP142-IHC assay (McNemar-Bowker analysis: *p* = 0.001) (Table 2). When using 50% PD-L1-positive TCs as the cut-off value, more than 50% of cells showing PD-L1 expression by the 22C3-IHC assay was considered as positive expression, and this was used as true positive results. The overall sensitivity and specificity of the SP142-IHC assay were 35% and 100%, respectively. When using 1% PD-L1-positive TCs as the cut-off point value, the SP142-IHC assay demonstrated a sensitivity of 57.8% and a specificity of 100%.

**Table 2:** PD-L1 IHC scoring according to the 22C3-IHC assay scoring algorithm.

		SP142-IHC assay			Total	Kappa value *P* value	McNemar-Bowker
		0	1+	2+			
22C3-IHC assay	0	89	1	0	90	0.481*P* < 0.001	*P* < 0.001
	1+	16	9	0	25		
	2+	4	9	7	20		
	Total	109	19	7	135		

0 indicates negative, 1 + weakly positive, and 2 + strongly positive PD-L1 expression.

With the adoption of the SP142-IHC assay scoring algorithm, 10 (7.4%), 14 (10.4%), 37 (27.4%) and 74 (54.8%) of 135 samples evaluated using the SP142-IHC the assay were classified into groups that were strongly positive, moderately positive, weakly positive, and negative for PD-L1 expression, respectively. However, only 69 (51.11%) of 135 samples produced the same results with the 22C3-IHC assay (κ value: 0.324, *p* < 0.001). In 60 (91.0%) of 66 discordant samples, PD-L1 status determined with the SP142-IHC assay was overestimated in the 22C3-IHC assay. In contrast, the PD-L1 status was underestimated by 22C3-IHC assay in six cases (McNemar-Bowker analysis: *p* = 0.001) (Table 3).

**Table 3:** PD-L1 IHC scoring according to the SP142-IHC assay scoring algorithm.

		SP142-IHC assay				Total	Kappa value *P* value	McNemar-Bowker
		0	1+	2+	3+			
22C3-IHC assay	0	40	5	0	0	45	0.481*P* < 0.001	*P* < 0.001
	1+	21	15	0	0	36		
	2+	9	11	5	1	26		
	3+	4	6	9	9	28		
	Total	74	37	14	10	135		

0 indicates negative, 1+ weakly positive, 2+ moderately positive, and 3+ strongly positive PD-L1 expression.

### Association of PD-L1 Expression with Clinicopathological Characteristics

**Figure 4:**
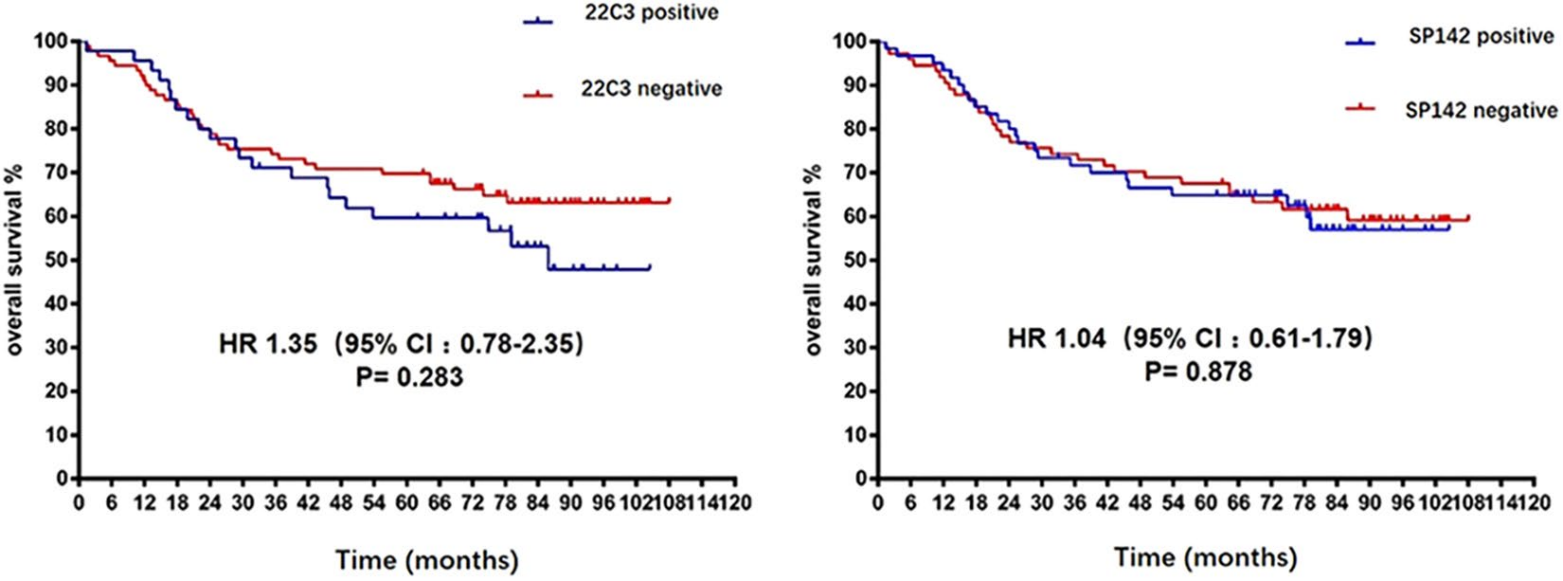
Kaplan–Meier curves showing correlation between the OS of patients and PD-L1 expression determined by the 22C3-IHC and SP142-IHC assays.

We determined the association of clinicopathological characteristics with PD-L1 expression based on each assay. PD-L1 expression based on the 22C3-IHC assay was significantly correlated with smoking status. However, the results from the SP142-IHC assay were different, indicating that PD-L1 expression was significantly correlated not only with smoking status but also with sex and histology (Table 4). Next, we evaluated the prognostic value of PD-L1 expression. No significant difference in survival was observed among patients with different PD-L1 expression by either assay **(**Fig. 4 **)**.

**Table 4:** Association of Clinicopathological Features with PD-L1 Expression Based on the 22C3-IHC and SP142-IHC assays.

Patient demographics (*N*=135)			22C3		SP142		
			Negative *N* (%)	*P* value	Positive *N* (%)	Negative *N* (%)	*P* value
Age (years)	>60 y	15 (11.1)	37 (27.4)	0.247	41 (30.4)	11 (8.1)	0.000
	≤60 y	30 (22.2)	53 (39.3)		20 (14.8)	63 (46.7)	
Sex	Male	36 (26.7)	62 (45.9)	0.221	52 (38.5)	46 (34.1)	0.003
	Female	9 (6.7)	28 (20.7)		9 (6.7)	20 (20.7)	
Smoking status	Never smoker	19 (14.7)	53 (41.1)	0.027	25 (19.4)	47 (36.4)	0.001
	Smoker	26 (20.2)	31 (24)		36 (27.9)	21 (16.3)	
Histology	SCC	24 (17.8)	29 (21.5)	0.053	35 (25.9)	18 (13.3)	0.001
	AD	18 (13.3)	49 (36.3)		22 (16.3)	45 (33.3)	
	Other subtypes	3 (2.2)	12 (8.9)		4 (3.0)	11 (8.1)	
TNM stage	I	22 (16.3)	40 (29.6)	0.557	36 (26.7)	26 (19.3)	0.418
	II	11 (8.1)	16 (11.9)		12 (8.9)	15 (11.1)	
	III	11 (8.1)	30 (22.2)		22 (16.3)	19 (14.1)	
	IV	1 (0.7)	4 (3.0)		4 (3.0)	1 (0.7)

## Discussion

In this study, we use two clinically validated PD-L1 antibodies, 22C3 and SP142, and the corresponding IHC assay platforms to assess both the reproducibility/concordance and heterogeneity of PD-L1 protein expression. The results showed that the SP142-IHC assay was associated with significantly lower levels of PD-L1 detection in both TCs and ICs than was the 22C3-IHC assay in most of the cases, indicating that assays using the SP142 and 22C3 antibodies cannot replace each other for the stratification of patients for pembrolizumab or atezolizumab-based immune therapy. However, if the SP142-IHC assay results are positive and the 22C3-IHC assay results are negative, we can trust the interpretation.

Many studies have explored the topic regarding assay compatibility, but the conclusions have been inconsistent. One study demonstrated an analytical equivalence of TC membrane labeling between three validated, commercially available PD-L1 IHC diagnostic assays that use three different monoclonal antibodies, namely, Ventana SP263 with durvalumab, Dako 22C3 with pembrolizumab, and Dako 28-8 with nivolumab. Another study was designed to assess interobserver concordance and PD-L1 IHC staining patterns. Fifteen resected pulmonary carcinoma specimens were centrally stained with the 28-8, 22C3, SP142, and SP263 IHC assays according to clinical trial protocols^12^. The data indicated that TCs can be reproducibly scored for PD-L1 expression based on IHC analysis in NSCLC tumors. No differences in interobserver concordance were observed among the examined assays, although the scoring of ICs had low concordance rates. However, that study did not include enough samples to be conclusive.

The Blueprint study was designed to evaluate the agreement of results from four PD-L1 IHC assays, namely, Dako 28-8, Dako 22C3, 29 Ventana SP263, and Ventana SP142. Thirty-eight non-interventional NSCLC cases were enrolled into the study, and each tumor was probed for PD-L1 using all four assays then scored by three pathologists. For TC staining, Dako 22C3, Dako 28-8 and Ventana SP263 assays demonstrated similar analytical performance, whereas Ventana SP142-IHC assay consistently labeled fewer TCs. Similar to our results, the SP142-IHC assay was associated with significantly lower levels of PD-L1 detection than was the 22C3-IHC assay^13^. However, that study was different from ours, as the authors evaluated the percentage of ICs that were positively stained, while we evaluated positive staining in ICs based on the percentage of tumor area. The study performed by Rimm *et al*. to compare the performance of 4 PD-L1 assays (28-8, 22C3, SP142, and E1L3N) indicated that the assay using the SP142 antibody differed from the others, as it detected significantly lower PD-L1 expression in TCs and ICs. The limitation of the study is that the authors used the SP142 antibody on a Ventana platform not identical to the platform now approved by the FDA^14^. In addition, the analysis was based on the average value as the gold standard to determine the sensitivity and specificity of various reagents. We used the standard for the 22C3-IHC as the gold standard with a 50% and 1% cut-off values and evaluated the sensitivity and specificity of PD-L1 labeling. We learned that the sensitivity with the SP142-IHC assay is poor but that the specificity is better. Thus, if the SP142-IHC assay results are negative, the 22C3-IHC assay results will also be negative. Because the details of antibodies are proprietary information, we can only speculate that the poor concordance of the results with the two antibodies may be due to different antibody affinities, limited specificity, or distinct target epitopes.

It is important to note that we employed standardized platforms and reagents that are now approved by the FDA for this study and that this is currently the largest clinical study assessing the concordance between SP142-IHC and 22C3-IHC results in NSCLC.

The prognostic impact of PD-L1 protein expression and its association with clinicopathological features have not been conclusively elucidated^15,16^. The reason for these discrepancies may be multifactorial^17^. Differences in distributions of histologic subtypes, antibodies and cut-off values may account for some of these discrepancies^18^. In our study, PD-L1 expression based on the 22C3-IHC assay was significantly correlated with smoking status. However, these results were different those obtained using the SP142-IHC assay, which indicated correlations of PD-L1 expression with sex, smoking status, and histology. Additionally, PD-L1 expression based on either IHC systems was not associated with patient prognosis. Our data represent the most direct evidence that demonstrates differences between two reagents and may partly explain the conflicting results regarding the correlation of PD-L1 expression and clinicopathological characteristics in other studies.

Our study has several limitations. First, all samples were obtained from patients who had not undergone PD1 or PD-L1 therapy. We did have access to therapeutic outcome data, and we could only evaluate the assay results by themselves without clinically meaningful comparisons of PD-L1 assays. Second, the vast majority of specimens were archived samples and not biopsies; these samples may not reflect the real situation in the clinic.

In conclusion, we identified that the use of different reagents for testing PD-L1 expression can be misleading; the two reagents cannot be interchanged with each other. Our findings also suggest that different reagents may account for inconsistent associations between the expression of PD-L1 and clinicopathological features.
